# Fully automatic segmentation of heart chambers in cardiac MRI using deep learning

**DOI:** 10.1186/1532-429X-18-S1-P351

**Published:** 2016-01-27

**Authors:** MR Avendi, Arash Kheradvar, Hamid Jafarkhani

**Affiliations:** 1 grid.266093.80000000106687243Center for Pervasive Communications and Computing, University of California, Irvine, Irvine, CA USA; 2 grid.266093.80000000106687243The Edwards Lifesciences for Advanced Cardiovascular Technology, University of California, Irvine, Irvine, CA USA

## Background

Cardiac magnetic resonance imaging (MRI) is now routinely being used for the evaluation of the function and structure of the cardiovascular system. Chamber segmentation from cardiac MRI datasets is an essential step for the computation of clinical indices such as ventricular volume, ejection fraction, mass and wall thickness as well as analysis of wall motion abnormality.

Manual delineation by experts is currently the standard clinical practice for performing chamber segmentation. However, manual segmentation is tedious, time consuming and prone to intra- and inter-observer variability. Therefore, it is necessary to reproducibly automate this task to accelerate and facilitate the process of diagnosis and follow-up.

Due to the several technical difficulties, automatic chamber segmentation from cardiac MRI dataset is still a challenging problem. Current automated techniques suffer from poor robustness and accuracy and require large training datasets and user interaction.

## Methods

**Figure 1:**
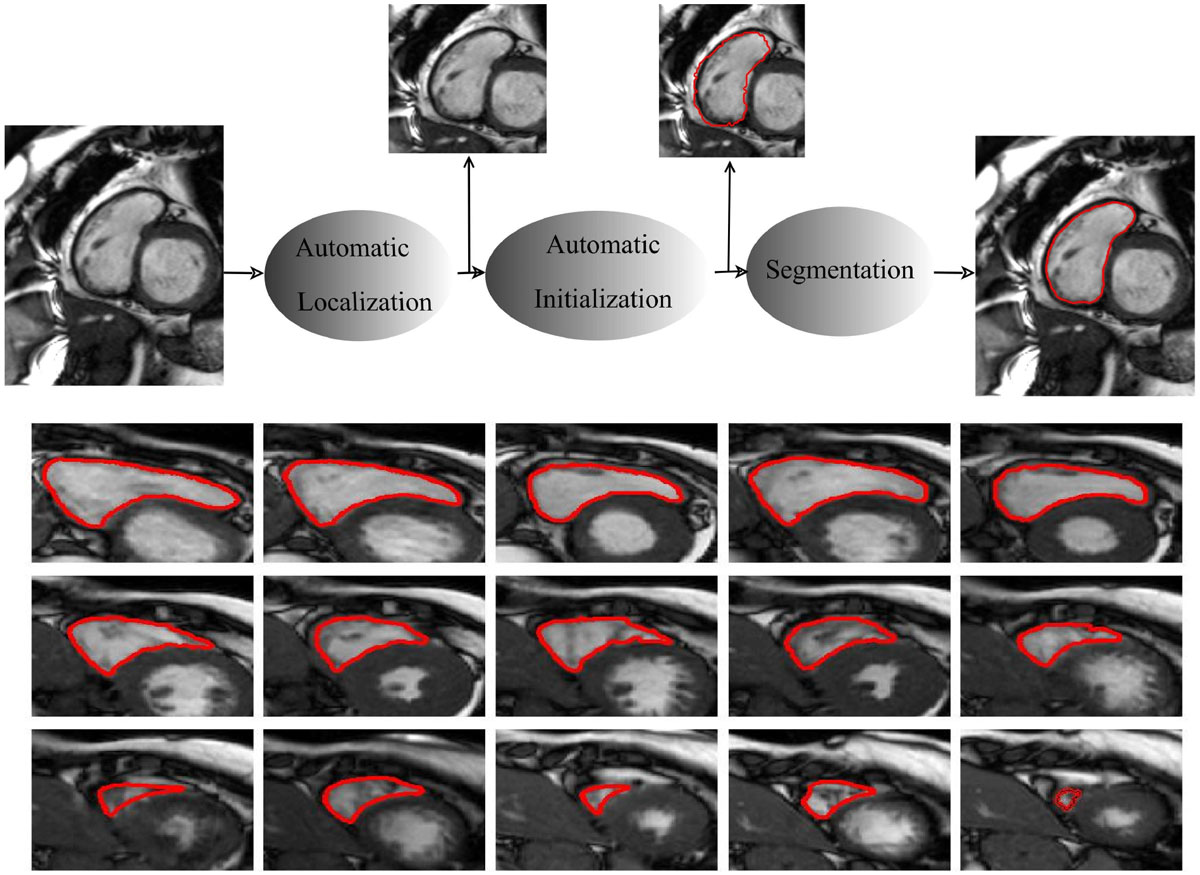
**(a): (top) Block diagram of the developed method for fully automatic chamber segmentation from cardiac MRI datasets (here RV is highlighted)**. Figure1 (b): (bottom) Automatic segmentation results of the RV from the base to the apex for a typical dataset of the MICCAI 2012 RV challenge.

In this study we developed and evaluated an accurate, fast and robust method for the fully automatic segmentation of the right ventricle (RV) and the left ventricle (LV) from cardiac MRI datasets. We tackled the complex problem of RV/LV segmentation by mimicking the function of human brain using deep learning. Deep learning is a branch of machine learning that aims to learn abstract concepts from high-dimensional data using multiple-layer computational models. Our method is composed of three stages (Figure 1a). First, the region of interest (ROI) containing the heart chamber is determined in the raw input image using deep convolutional networks trained to locate the chamber. Then, the chamber is initially segmented using a stacked autoencoder trained to delineate the chamber. The obtained contour is used as initialization and also is incorporated into deformable models for accurate, fast and robust segmentation. We utilized a portion of available MRI databases to train our method and then evaluated our method on the rest of the databases. The MICCAI 2012 RV segmentation challenge database and the MICCAI 2009 LV database, were used in the RV and LV segmentation studies, respectively.

## Results

Good agreement with the ground truth was obtained. Quantitative results for RV segmentations are summarized in Table 1. Illustrative results for a typical dataset are illustrated in Figure 1b. Also, for the LV, a correlation with the ground truth contours of 0.99, 0.99, 0.99 for ED volume, ES volume and Ejection Fraction was measured.

**Table 1:** Comparison of RV segmentation results of our method and the state-of-the-art methods tested on MICCAI 2012 database.

Method	A/SA	DM	HD(mm)	R(EDV)	R(ESV)	R(EF)
Our Method	A	0.81(0.21)	7.79(5.91)	0.99	0.99	0.95
CMIC (Zuluaga et al., 2013)	A	0.78(0.23)	10.51(9.17)	0.93	0.93	0.73
NTUST (Wang et al., 2012)	SA	0.57(0.33)	28.44(23.57)	0.71	0.78	0.23
SBIA (Ou et al., 2011)	SA	0.55(0.32)	23.16(19.86)	0.63	0.69	0.45
BIT-UPM (Maier et al., 2012)	SA	0.80(0.19)	11.15(6.62)	0.99	0.97	0.84
GEWU(Nambakhsh et al., 2013)	SA	0.59(0.24)	20.21(9.72)	0.81	0.81	0.4
ICL(Bai et al., 2013)	SA	0.78(0.2)	9.26(4.93)	0.98	0.98	0.85
LITIS(Grosgeorge et al., 2013)	SA	0.76(0.2)	9.97(5.49)	0.95	0.90	0.76

A: Automatic, SA: semi-Automatic. Hausdorff Distance (HD), Dice Metric (DM), correlation coefficient (R).

## Conclusions

Chamber segmentation from MRI datasets can be fully automated using our deep learning approach. The proposed method outperforms the state-of-the-art methods in terms of accuracy, robustness and computational time. The high correlation between the automatic and manual references shows the accuracy and clinical applicability of the proposed framework for automatic evaluation of the heart function.
